# Congenital Pseudarthrosis of the Clavicle: A Case Report and a Hypothesis for the Right-Side Predominance

**DOI:** 10.7759/cureus.29157

**Published:** 2022-09-14

**Authors:** Sameer Y Al-Abdi, Ali M Khalil, Amr N Elnamky, Karim E Sherif

**Affiliations:** 1 Neonatology, King Abdulaziz Hospital, Al-Ahsa, SAU; 2 Neonatology, Al Ahsa Hospital, Al-Ahsa, SAU

**Keywords:** newborn, congenital pseudarthrosis of the clavicle, clavicle, pseudarthrosis, congenital

## Abstract

Congenital pseudarthrosis of the clavicle (CPC) is rare. It predominantly affects the right side for an unknown reason. Most of the reported cases are diagnosed outside the neonatal period. Only two CPC cases have been reported in Saudi Arabia, where both were diagnosed during childhood. Here, we present the case of a Saudi male newborn with right-sided CPC. The diagnosis was made shortly after birth because of the uneventful cesarean delivery and painless clavicular lump. Fetuses prefer keeping their head in a right lateral position which may be a plausible explanation for the right-side predominancy in the CPC.

## Introduction

Congenital pseudarthrosis of the clavicle (CPC) is a rare condition with an unknown etiology. It affects males and females with a ratio of 1:1 during the last decades [1]. It predominantly affects the right side for an unknown reason. About 323 cases have been reported worldwide [1-3]. Most reported cases were diagnosed outside the neonatal period [1,4]. Only two CPC cases have been reported in Saudi Arabia, where both were diagnosed during childhood [2,3]. The CPC is an important differential diagnosis for the common acute clavicular fracture from birth trauma [1,4]. However, the CPC is still not well addressed in neonatology textbooks [5]. Thus, we report a Saudi male newborn diagnosed with unilateral right-sided CPC shortly after birth to increase awareness of this condition among newborn healthcare professionals. We suggest a new hypothesis for the right-sided predominance of the CPC.

## Case presentation

**Figure 1:**
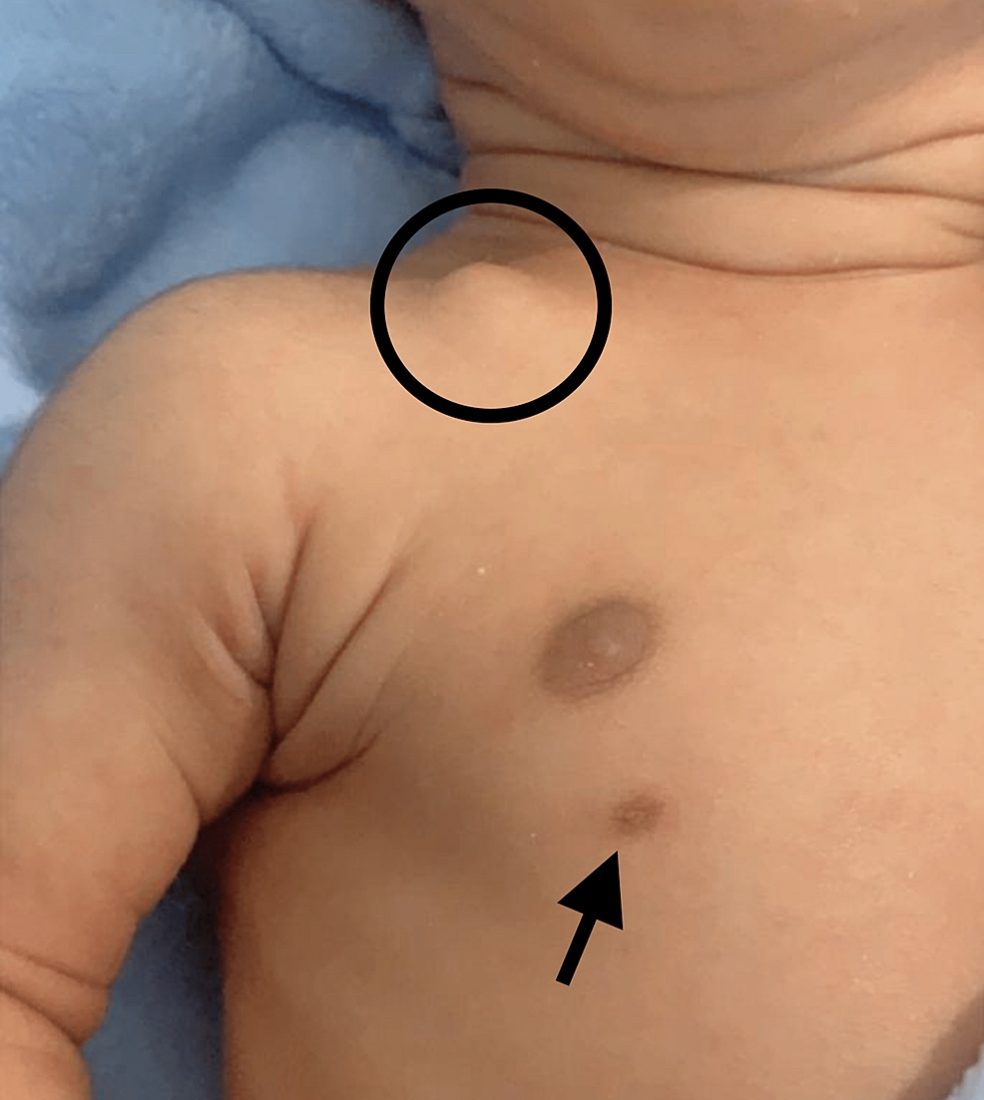
Lump of congenital pseudarthrosis of the right clavicle (circle) and supernumerary nipple (arrow) in a newborn.

**Figure 2:**
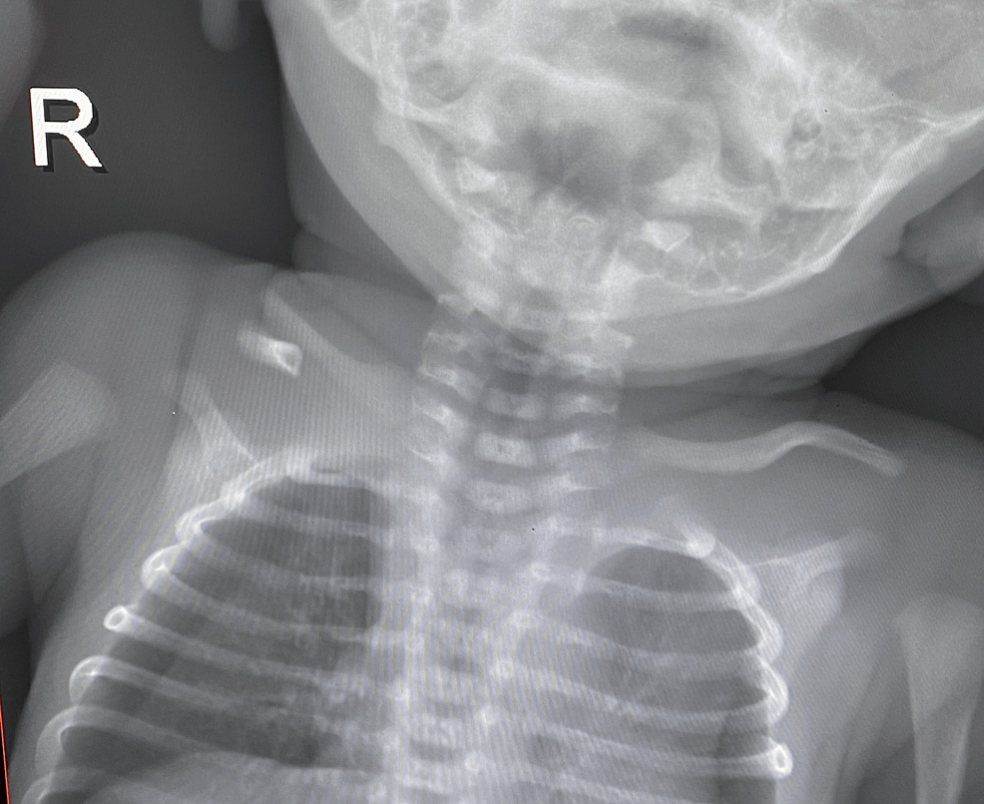
X-ray demonstrating congenital pseudarthrosis of the right clavicle.

A cephalic male newborn twin was born by cesarean section at 36 + 4/7 weeks of gestation to a primigravida Saudi mother. His birth weight was 2,325 g which was appropriate for gestation age as it was above the 10th percentile of Fenton’s growth charts [6]. The delivery was uneventful, with no history of trauma. Shortly after birth, he was noticed to have a bony lump over the middle third of the right clavicle (Figure 1). The swelling was painless, mobile, and with rounded edges. The skin over the lump and the whole body was normal except for a unilateral right-sided supernumerary nipple (Figure 1). A posterior-anterior chest X-ray revealed the medial fragment of the right clavicle positioned above the lateral piece without any callous formation (Figure 2). The X-ray raised the possibility of a right-sided pneumothorax. However, a left lateral decubitus X-ray of the chest showed no pneumothorax, and the baby was completely asymptomatic from a respiratory point of view.

The newborn was referred to the orthopedic service and the plan was to only observe. The clinical condition of the newborn was static when he was seen in our clinic at two weeks of age. The other twin was also male but was unaffected.

## Discussion

We present the youngest reported case of unilateral right-sided CPC in Saudi Arabia. The diagnosis was made shortly after birth because delivery was by cesarean and the clavicular lump was painless.

Acute clavicular fracture from birth trauma is the most common differential diagnosis of CPC. Our case is non-traumatic for several reasons. First, the delivery was an uneventful cesarean section. Second, the lump was painless. Third, an X-ray showed sclerotic closure of the medullary canal without forming reactional bone calluses. Fourth, the clinical and radiological findings of our case are typical of CPC because it was unilateral right-sided involvement, and the medial fragment of the right clavicle was positioned above the lateral piece [1,4].

Unilateral involvement of the right clavicle is one of the cardinal features of the CPC [1,4]. However, it is still mysterious why the right clavicle is much more affected than the left. As the right subclavian artery is at a higher position than the left one, it has been proposed that a combination of abnormal fetus position and pressure effect of subclavian artery pulsation interfere with clavicle development [4]. Indeed, normally fetuses prefer keeping their head in the right lateral position from 32 weeks gestation onwards [7,8]. This fetal right lateral head position preference increases as gestational age increases [7,8]. Thus, we hypothesize that the normal fetal right lateral head position preference may be a plausible explanation for the right-sided predominancy as it may augment the external pressure on the right clavicle. However, our hypothesis still needs to be confirmed.

A 2012 systemic review suggested that only the left-sided CPC is associated with other congenital anomalies [4]. However, the 2022 systemic review has not confirmed this association [1]. Our case has a unilateral right-sided supernumerary nipple. As supernumerary nipple is a common minor congenital anomaly [9], we believe that the coexistence of the right-sided CPC and polythelia in our case is just accidental.

## Conclusions

The CPC is a rare pathology that needs a high suspicion index to diagnose during the neonatal period. The fetal right lateral head position preference may be a plausible explanation for the right-sided predominance of CPC.
